# Multi-Layer Palladium Diselenide as a Contact Material for Two-Dimensional Tungsten Diselenide Field-Effect Transistors

**DOI:** 10.3390/nano14050481

**Published:** 2024-03-06

**Authors:** Gennadiy Murastov, Muhammad Awais Aslam, Simon Leitner, Vadym Tkachuk, Iva Plutnarová, Egon Pavlica, Raul D. Rodriguez, Zdenek Sofer, Aleksandar Matković

**Affiliations:** 1 Department Physics, Mechanics and Electrical Engineering, Montanuniversität Leoben, Franz Josef Strasse 18, 8700 Leoben, Austriasimon.leitner@stud.unileoben.ac.at (S.L.); 2 Laboratory of Organic Matter Physics, University of Nova Gorica, Vipavska 13, SI-5000 Nova Gorica, Slovenia; vadym.tkachuk@ung.si (V.T.); egon.pavlica@ung.si (E.P.); 3 Department of Inorganic Chemistry, University of Chemistry and Technology Prague, Technická 5, 166 28 Prague, Czech Republic; plutnari@vscht.cz (I.P.); zdenek.sofer@vscht.cz (Z.S.); 4 Research School of Chemistry & Applied Biomedical Sciences, Tomsk Polytechnic University, Lenina ave. 30, 634034 Tomsk, Russia; raul@tpu.ru

**Keywords:** palladium diselenide, tungsten diselenide, tungsten selenium oxide, semi-metal, laser treatment, contact engineering, field-effect transistor, pMOS, van der Waals electronics, 2D materials

## Abstract

Tungsten diselenide (${\mathrm{WSe}}_{2}$) has emerged as a promising ambipolar semiconductor material for field-effect transistors (FETs) due to its unique electronic properties, including a sizeable band gap, high carrier mobility, and remarkable on–off ratio. However, engineering the contacts to ${\mathrm{WSe}}_{2}$ remains an issue, and high contact barriers prevent the utilization of the full performance in electronic applications. Furthermore, it could be possible to tune the contacts to ${\mathrm{WSe}}_{2}$ for effective electron or hole injection and consequently pin the threshold voltage to either conduction or valence band. This would be the way to achieve complementary metal–oxide–semiconductor devices without doping of the channel material.This study investigates the behaviour of two-dimensional ${\mathrm{WSe}}_{2}$ field-effect transistors with multi-layer palladium diselenide (${\mathrm{PdSe}}_{2}$) as a contact material. We demonstrate that ${\mathrm{PdSe}}_{2}$ contacts favour hole injection while preserving the ambipolar nature of the channel material. This consequently yields high-performance *p*-type ${\mathrm{WSe}}_{2}$ devices with ${\mathrm{PdSe}}_{2}$ van der Waals contacts. Further, we explore the tunability of the contact interface by selective laser alteration of the ${\mathrm{WSe}}_{2}$ under the contacts, enabling pinning of the threshold voltage to the valence band of ${\mathrm{WSe}}_{2}$, yielding pure *p*-type operation of the devices.

## 1. Introduction

Two-dimensional (2D) materials, and especially 2D semiconductors, are emerging as ever-more promising platforms to be added into very-large-scale integration (VLSI) technologies [1,2]. This is driven by the shrinking pitch sizes required to achieve higher integration density, energy efficiency, and speed of electronic circuits [3,4]. To achieve this feat, comprehensive studies have been undertaken to offer 2D channel materials and insulators [5,6] with performance parameters comparable to those of silicon-based technologies. Both traditional (${\mathrm{Al}}_{2}$$O_{3}$${\mathrm{/HfO}}_{2}$) and 2D insulators have shown promising results in developing complex architectures [7,8]. Similarly, a huge library of 2D semiconductors is available for the purpose of choosing *p*-type, *n*-type, or ambipolar channel materials [9,10]. In this regard, ambipolar ${\mathrm{WSe}}_{2}$ has garnered keen interest in the scientific community due to its potential applications towards complementary metal–oxide–semiconductor (CMOS) technology, solar cells, water splitting, light emitting, and gas sensing [11,12,13,14,15]. Moreover, patterned nanoribbons of ${\mathrm{WSe}}_{2}$ have been shown to offer high electrical performance and the possibility to be coupled with metallic nanoparticles, which offers exciting possibilities in optoelectronic applications and tunable catalysis [16,17]. Like other 2D materials, the properties of ${\mathrm{WSe}}_{2}$ can be tuned via thickness [18], plasma treatment [19], strain [20], and choice of contacts [21,22].

However, the development of technology-relevant metal–semiconductor interfaces remains a significant bottleneck for the integration of 2D semiconductors into VLSI [1,23]. This is also true for achieving high-quality contacts to ambipolar ${\mathrm{WSe}}_{2}$. The existing metal electrode deposition technologies cause the degradation of the 2D materials at the contact interface by the formation of metal-induced gap states (MIGS) and defect-induced gap states. In turn, these gap states result in the formation of large barriers at the junctions, consequently lowering the device performance and increasing energy consumption [24,25]. In addition to the creation of potential barriers, MIGS also alter transport fundamentally by changing transmission around the transport gap. An example of this can be seen in metallization-induced change of the quantum limits of contact resistance in one-dimensional contacts to semiconducting graphene nanoribbons [26]. There have been several efforts to find suitable contact materials and contact deposition methods to realise the full potential of 2D material-based circuits. These include the use of edge contacts [27,28], low-work-function metals [29], ultra-high vacuum evaporation [30], buffer layers [31], self-assembled dipolar monolayers [32], and dry stamping of metal electrodes [33,34]. In particular, metallised edge contacts are commonly the best-performing technology in large-area 2D material-based devices [27,28]. However, this is not the case for one-dimensional (1D) or quasi-1D nanostructures of 2D materials such as nanoribbons and nanowires, as phosphorene nanodevices with edge contacts [35,36].

More recently, semi-metallic contacts such as bismuth, antimony, and graphene have shown promising results [37,38,39]. However, semi-metal depositions involve heating of the substrate up to 100 °C to achieve a particular orientation of the metal (Sb 011$\bar{2}$ on ${\mathrm{MoS}}_{2}$), which tends to introduce defects into heat-sensitive 2D semiconductors with ambipolar functionality, such as ${\mathrm{WSe}}_{2}$ [40,41] and black phosphorus [42]. In case of graphene contacts, the growth of graphene films as top electrodes would require very high temperatures [43], hindering direct growth as the incorporation path to the back-end-of-line (BEoL) processes. However, ambipolar 2D materials are critical for CMOS electronic applications. It is essential to develop contacts which preserve the ambipolar behaviour while providing sufficient on-state currents and $I_{\mathrm{ON}}$/$I_{\mathrm{OFF}}$ ratios relevant for technological aspects. To address this challenge, significant efforts have been made, including the use of In and Pd contacts to ${\mathrm{WSe}}_{2}$ [44,45].

The use of ${\mathrm{PdSe}}_{2}$ as a contact material was first demonstrated by Oyedele et al., who employed defective ${\mathrm{Pd}}_{17}$${\mathrm{Se}}_{15}$ as contacts with ${\mathrm{PdSe}}_{2}$ to demonstrate a low Schottky barrier [46] and later by Seo et al. for the realization of ${\mathrm{PdSe}}_{2}$-based CMOS devices [47]. ${\mathrm{PdSe}}_{2}$ has also been used to contact ${\mathrm{MoS}}_{2}$ in a junction field-effect transistor as a top gate due to its promising optoelectronic properties which include long-wavelength infrared photo responsivity [48,49,50,51]. With a layer-dependent bandgap in the infrared region, ${\mathrm{PdSe}}_{2}$ itself is a unique member of the transition metal dichalcogenide family with potential uses in optoelectronic devices [52]. It behaves as a semi-metal for thicknesses above 20 nm and transforms to a semi-conducting state for thinner layers [53,54]. Large-area ${\mathrm{PdSe}}_{2}$ can be grown at temperatures as low as 250 °C [55], unlike graphite, which makes it critical for BEoL integration as a van der Waals electrode material. Moreover, graphite contacts dope the ${\mathrm{WSe}}_{2}$ towards a dominant *n*-type electrical response, therefore disrupting the ambipolar nature of ${\mathrm{WSe}}_{2}$ [56].

In this work, we propose ${\mathrm{PdSe}}_{2}$ contacts to ${\mathrm{WSe}}_{2}$ which demonstrate high $I_{\mathrm{ON}}$/$I_{\mathrm{OFF}}$ ratio and high on-state currents while maintaining the intrinsic ambipolar behaviour of the channel material. To further tune the behaviour of our devices, we propose localised laser treatment of ${\mathrm{WSe}}_{2}$ at the contact regions to demonstrate dominant *p*-type FETs with high threshold voltage stability. This can allow for the co-integration of *p*-type and ambipolar devices in a circuit without the need to change the contact material nor the need to introduce any dopants into the channel.

## 2. Materials and Methods

### 2.1.${\mathrm{PdSe}}_{2}$ Crystal Growth

${\mathrm{PdSe}}_{2}$ crystals were synthesised by direct reaction of elements in a quartz glass ampoule. Powder palladium (99.99%, −100 mesh, Safina, Prague-Vestec, Czech Republic) and selenium granules (99.9999%, 2–4 mm granules, Wuhan Xinrong New Material Co., Wuhan, China) corresponding to 3 g of ${\mathrm{PdSe}}_{2}$ were placed in a quartz ampoule (25 × 100 mm) with additional selenium corresponding to 1% in excess. The ampoule was melt sealed under a high vacuum (1 × $10^{-3}$ Pa) using an oxygen–hydrogen torch and placed in muffle furnace. The ampoule was heated at 850 °C using a heating rate of 1 °C/min, and after 12 h it was cooled to room temperature at a cooling rate of 0.1 °C/min. The ampoule with formed ${\mathrm{PdSe}}_{2}$ crystals was opened in an argon-filled glove box.

### 2.2. Device Fabrication

Using laser lithography (DaLi, Cerklje, Slovenia) and thermal evaporation, 45 nm/5 nm stripe-like Au/Cr electrodes were patterned onto a 300 nm ${\mathrm{SiO}}_{2}$/Si substrate. hBN flakes were used as a bottom gate oxide on top of one the Au pads. Multi-layer ${\mathrm{PdSe}}_{2}$ or crystal (kish) graphite flakes were placed on top of the ${\mathrm{WSe}}_{2}$ flakes as source and drain electrodes. Flakes of 2D materials were mechanically exfoliated from bulk single crystals using commercially available Nitto tape and polydimethylsiloxane (PDMS) Gel–Pak–DGL–X4. The flakes were selected based on optical contrast and transferred one by one to build up the devices. The thickness of the hBN used for the devices was approximately 20 nm, considering a value for the relative dielectric constant of $\epsilon_{r}$ = 3.5 [57]. Further, optical microscopy, atomic force microscopy (AFM), and Raman spectroscopy measurements were performed to confirm the layer thickness, uniformity, and exact device geometries.

### 2.3. Electrical Characterization

Room temperature (RT) and low-temperature (78 K) electrical characterizations were performed using a Keithley 2636A Source Meter (Tektronix GmbH, Koeln, Germany) attached to an Instec probe station (Boulder, CO, USA). The samples were contacted via Au-coated Ti electrical cantilever microprobes. The Instec’s mK2000 temperature controller was used to monitor the temperature with a resolution of 0.01 K. The cooling and heating rates were 20 °C/min and 10 °C/min, respectively.

### 2.4. FET Figures of Merit (FOM) Extraction and Device Modelling

The off-state current ($I_{\mathrm{OFF}}$) was defined as the minimum in $I_{D}\left(V_{G}\right)$ curves, while the on-state current ($I_{\mathrm{ON}}$) was defined as the maximum obtained in $I_{D}\left(V_{G}\right)$ for the electron or hole branch. The maximum current is limited by the amount of the electrostatic field that can be applied through the back gate, and $I_{\mathrm{ON}}$ was estimated 5 to 8 V away from the threshold voltage ($V_{\mathrm{th}}$). The threshold voltage was estimated by extrapolation of the linear fit to the point of intersection of the $I_{D}$ = 0 A line. The linear fit was performed in the $V_{G}$ region shifted by 2 V from the onset voltage point ($V_{\mathrm{on}}$) and by 4 V to 5 V from the $V_{\mathrm{on}}$. The onset voltage point was defined as the $V_{G}$ point from which the $I_{D}$ continuously increases from the gate leakage levels (usually 0.5–2 ×$10^{-11}$ A). The middle of the region between $V_{\mathrm{on}}$ and $V_{\mathrm{th}}$ was used to estimate the sub-threshold swing (SS) values.

Modelling of the FET output curves was performed using the ideal transistor operating in the linear regime, shifted by the $V_{\mathrm{th}}$ via a capacitor at the gate. To model the non-ideal and non-linear behaviour of the contacts, a linear resistor and a Schottky diode were added in series to the ideal transistor. The current through the transistor was described as: $I_{D}=\left(\mu C_{\mathrm{ox}}W/L\right)\cdot \left(\left(V_{G}-V_{\mathrm{th}}\right)\cdot V_{\mathrm{FET}}+\left(V_{\mathrm{FET}}^{2}/2\right)\right)$. Here, $V_{\mathrm{FET}}$ corresponds to the fraction of the total $V_{D}$ bias that is experienced by the ideal transistor, $C_{\mathrm{ox}}$ is the area-specific gate dielectric capacitance, and μ is the intrinsic mobility. Upon reaching the limit of the linear regime — defined as the maximum $I_{D}\left(V_{\mathrm{FET}}\right)$ value of the model — the maximum current level was kept independent of the $V_{\mathrm{FET}}$, describing the saturation of the device. The ohmic component of the contact resistance is defined by a linear resistor, with its corresponding potential drop described as $V_{\mathrm{ohmic}}=R_{\mathrm{ohmic}}\cdot I_{D}$. The non-linear component of the contact resistance is described by: $V_{\mathrm{junction}}=V_{\mathrm{thermal}}\ln\left(1+I_{D}/I_{0}\right))$. Here, $V_{\mathrm{thermal}}=k_{B}T/e$ and $k_{B}$ stands for the Boltzmann’s constant, *T* is fixed to the set temperature of the experiment, and *e* is the unit charge. $I_{0}$ represents the reverse current of the Schottky diode. Considering that $V_{D}=V_{\mathrm{FET}}+V_{\mathrm{ohmic}}+V_{\mathrm{junction}}$ the system is solved in a self-consistent manner using three fitting parameters: μ, $R_{\mathrm{ohmic}}$, and $I_{0}$. In the first fitting iteration, the parameters are assumed to be independent of $V_{G}$ and are fitted to the sequence of the electrical output curves for the hole or the electron branch. In the second iteration, for each $V_{G}$ the parameters are allowed to vary by ±20% from the previously determined values. The contact resistance is further expressed as a device width-scaled (*W*) value: $WR_{C}=W\left(\left(V_{\mathrm{ohmic}}+V_{\mathrm{junction}}\right)/I_{D}\right)$.

### 2.5. Laser Treatment of  ${\mathrm{WSe}}_{2}$

The freshly exfoliated channel ${\mathrm{WSe}}_{2}$ was laser-treated (532 nm, 100× objective) under ambient conditions using a motorised sample stage. The laser modification of ${\mathrm{WSe}}_{2}$ was performed prior to the transfer of ${\mathrm{PdSe}}_{2}$ contacts. The laser power was set to 50 mW. A point-to-point scan was carried out with a resolution of 0.2 µm, and a fixed exposure time of 0.1 s for each point.

### 2.6. AFM and In Operando KPFM Measurements

Horiba/AIST-NT Omegascope (Lille, France) AFM system was used for the AFM topography measurements, with Nunano SPARK 350 Pt probes (spring constant of 42 ${\mathrm{Nm}}^{-1}$, resonant frequency 330 kHz, and tip radius of 30 nm). Topography images were processed in the open-source software Gwyddion v2.56 [58], applying zero-order line correction and three-point plane averaging.

In operando Kelvin Probe Force Microscopy (KPFM) measurements were carried out on ${\mathrm{PdSe}}_{2}$-contacted devices under the ambient conditions. To prevent device degradation during prolonged ambient operation, the devices for the KPFM experiments were top capped by an additional 10 nm thick hBN flake. For the device biasing during the KPFM measurements, a Keithley 2636A Source Meter was used, and the device ground (source) was connected to the ground of the KPFM feedback loop. KPFM was operated in a frequency-modulated two-pass regime with a second-pass lift height of 8 nm, yielding a total of about 18 nm distance between the probe and the hBN capped channel. To extract electrostatic potential drops across the channel of an operating device, a single line in the middle of the device was repeatedly scanned while the external bias was applied. To compensate for the work function and stray field differences, each potential drop is normalised to the cross-sections recorded with $V_{D}$ = 0 V, following the procedure detailed in Ref. [32].

### 2.7. Raman Spectroscopy

Raman spectroscopy measurements were performed using a Horiba LabRam HR Evolution confocal Raman spectrometer (Lille, France) with 1800 lines/mm gratings. A 532 nm laser was used with an excitation power in the range of 0.1–3.2 mW. The laser spot was focused by a 100×, 0.9 NA objective.

## 3. Results and Discussions

### 3.1. Electrical Characteristics of ${\mathrm{WSe}}_{2}$ FETs with Graphite and ${\mathrm{PdSe}}_{2}$ Electrodes

**Figure 1:**
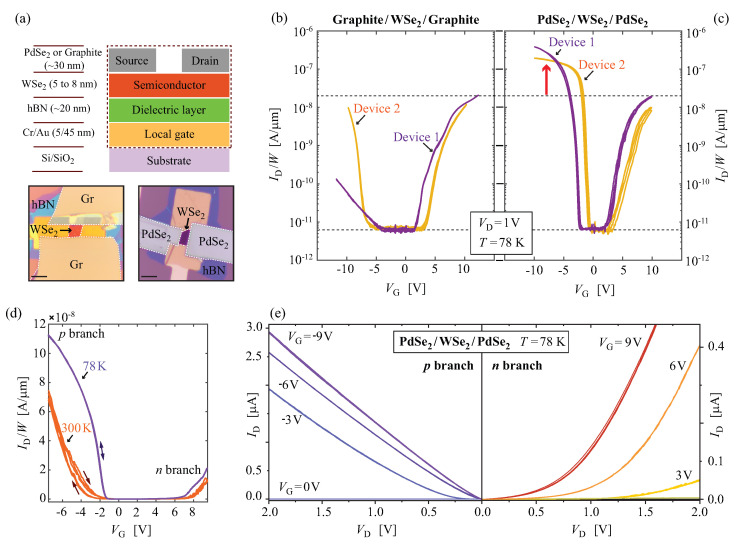
Electrical characteristics of graphite- and ${\mathrm{PdSe}}_{2}$-contacted ${\mathrm{WSe}}_{2}$ FETs: (**a**) Schematic representation of device configuration with optical images of ${\mathrm{WSe}}_{2}$ FETs (scale bar: 10 µm). (**b**,**c**) Semi-log electrical transfer curves of devices with graphite (Gr) and ${\mathrm{PdSe}}_{2}$ contacts, respectively. The $I_{D}$ in (**b**,**c**) is scaled by the mean width of the channels to allow for better comparison of the current values between the different devices. The horizontal dashed lines that interconnect (**b**,**c**) serve as a guide to see the reached on- and off-state current levels. The red arrow in (**c**) indicates over an order of magnitude larger current of the hole branch in the case of ${\mathrm{PdSe}}_{2}$ contacts. (**d**) Comparison of the device width-scaled electrical transfer curves (${\mathrm{PdSe}}_{2}$ contacted device) measured at 300 K (orange) and 78 K (purple), presented in linear scale. The arrows indicate the direction of the $V_{G}$ sweep, highlighting an increase in the hysteresis observed at 300 K. (**e**) Output curves for the hole and electron branches at 78 K (2 × $10^{-2}$ mbar) of a device with ${\mathrm{PdSe}}_{2}$ contacts. Note that the current values for the *n*-branch are approximately one order of magnitude lower than for the *p*-branch. The different colored lines in (**e**) represent the curves at the different values of $V_{G}$, as indicated in the figure.

Figure 1a depicts a schematic representation of the 2D layer stacks along with optical images of the typical devices with graphite (Gr) and ${\mathrm{PdSe}}_{2}$ contacts. Figure 1b,c present in a semi-log scale the device width-scaled electrical transfer curves, source–drain current as a function of the applied local back gate bias $I_{D}\left(V_{G}\right)$, comparing the two different types of van der Waals contacts (Gr and ${\mathrm{PdSe}}_{2}$) to multilayered ${\mathrm{WSe}}_{2}$. For each transfer curve, five subsequent forward and backward $V_{G}$ sweeps were carried out at 2 V/s. In both cases, a small hysteresis of 200 mV was observed. The measurements were carried out at 78 K to minimise charge-trap-related effects and unintentional doping effects from the trapped water and air at the interfaces [16,59,60]. Graphite-contacted devices showed a dominant *n*-type behaviour which was previously reported and attributed to the band alignment that favours electron injection from the graphite towards the channel material [56,61]. In Figure 1b, the second device (Device 2) also exhibits notably high current in the hole branch, however, the threshold voltage remains closer to the electron branch, as expected for the efficient electron injection from graphite electrodes [56].

The main difference in the electrical transfer curves between Gr and ${\mathrm{PdSe}}_{2}$-contacted devices occurs at the negative $V_{G}$ values, i.e., in the hole branch. In contrast to graphite-contacted devices, when ${\mathrm{PdSe}}_{2}$ is used as a contact, the FETs were found to exhibit dominant *p*-type behaviour and an increased device performance for both electron and hole branches. This is explained by the favoured level alignment of the ${\mathrm{PdSe}}_{2}$ with the hole branch of the ${\mathrm{WSe}}_{2}$ due to the higher work function of ${\mathrm{PdSe}}_{2}$ in comparison to graphite. The $I_{\mathrm{ON}}/I_{\mathrm{OFF}}$ ratio for ${\mathrm{PdSe}}_{2}$ (∼4 × $10^{4}$) was one order of magnitude better than that of graphite contacts. Horizontal dashed lines that interconnect Figure 1b,c serve as a guide to help compare the current levels. For the ${\mathrm{WSe}}_{2}$ devices reported in the literature, the $I_{\mathrm{ON}}/I_{\mathrm{OFF}}$ ratio varies over several orders of magnitude [11]: from $10^{2}$ (e.g., ${\mathrm{NbSe}}_{2}$ contacts to the *n*-branch [56]) up to $10^{9}$ with more elaborate device architectures and high-k dielectrics [18]. With respect to the electrode engineering to access the *p*-branch,  ${\mathrm{NbSe}}_{2}$- and Pt-contacted ${\mathrm{WSe}}_{2}$ were reported to reach the values in the range $10^{4}$–$10^{7}$ [18,56].

Furthermore, ${\mathrm{PdSe}}_{2}$-contacted devices maintained an intrinsic behaviour which is evident by an almost equidistant $V_{\mathrm{on}}$ for both electron and hole branches with reference to $V_{G}=$ 0 V. This was not the case for graphite-contacted devices where larger $V_{G}$ was required to reach the on state of the *p*-branch compared to the *n*-branch, therefore indicating a disruption in the intrinsic doping levels. On average, we observe a $V_{\mathrm{on}}$ for the hole branch to be at (−4.5 ± 0.9) V and at (−1.9 ± 1.3) V respectively for the Gr and ${\mathrm{PdSe}}_{2}$ contacts; similar values for the $V_{\mathrm{on}}$ were observed for the electron branch: (2.5 ± 1.7) V and (3.0 ± 0.8) V respectively for the Gr and ${\mathrm{PdSe}}_{2}$ contacts.

A comparison between 300 K and 78 K width-scaled transfer curves of a ${\mathrm{PdSe}}_{2}$-contacted device is presented in Figure 1d. The temperature primarily impacts the phonon-related carrier scattering in the channel, the Schottky junction-related potential drop, and gate dielectric interface charge trap states. Consequently, at lower temperatures we observe an overall increase in the drain currents and mobilities for both branches (by a factor of ∼2 comparing 78 K and 300 K), quenching of the hysteresis with respect to the forward and backward $V_{G}$ sweeping, and a minor reduction in the $V_{\mathrm{th}}$ values.

**Figure 2:**
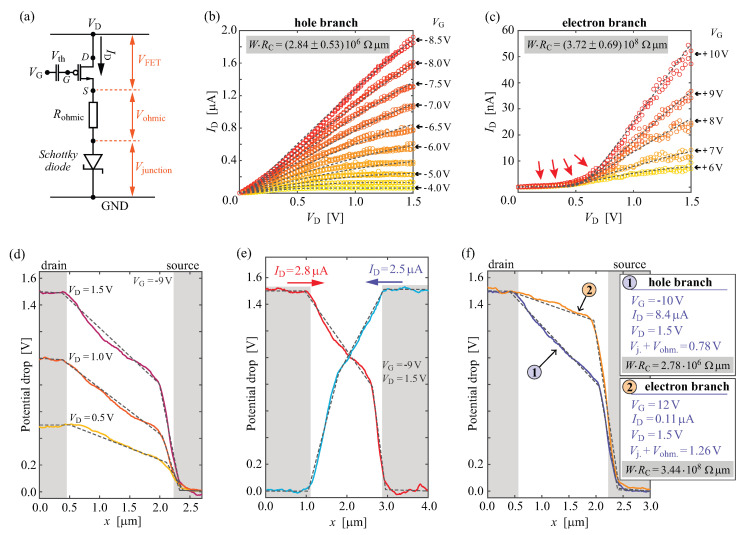
Contact resistance of the ${\mathrm{PdSe}}_{2}$${\mathrm{/WSe}}_{2}$ interface: (**a**) Equivalent electrical scheme used for the self-consistent modelling of the output curves. (**b**,**c**) Electrical output curves of a${\mathrm{PdSe}}_{2}$${\mathrm{/WSe}}_{2}$${\mathrm{/PdSe}}_{2}$ device measured at 300 K for the hole and electron branches, respectively. Different colored circles represent the measured $I_{D}$ values at set different $V_{G}$ as indicated in the right corner of the sub-panels (**b**,**c**). The dashed lines are a model for the entire data set. Red arrows in (**c**) indicate a severe downward bending of the output curves at lower $V_{D}$. Contact resistance values ($WR_{C}$) extracted by modelling the curves from (**b**,**c**) are indicated in each sub-panel. (**d**–**f**) In operando KPFM potential profiles recorded as single lines across the channel, measured under ambient conditions. Solid lines present the work function difference corrected potential drops, and the dashed lines are linear fits to the experimental curves. (**d**) A sequence of the potential drops with varied $V_{D}$. (**e**) Alternating the source and drain contacts, which demonstrates that the steep potential drop is related to the grounded electrode. (**f**) Comparison of the potential drops at $V_{D}$ = 1.5 V, with $V_{G}$ setting the device in an on state of the hole and electron branches, labelled with (1) and (2), respectively. Insets in (**f**) provide the operation points and the extracted $WR_{C}$ values from the KPFM measurements.

Figure 1e represents the electrical output curves for the ${\mathrm{PdSe}}_{2}$-contacted channel; the source–drain current as a function of the applied source–drain bias is $I_{D}$($V_{D}$). Especially at more negative $V_{G}$ values (on state of the *p*-branch), the electrical output curves of the *p*-branch exhibit linear behaviour. For the *n*-branch, the overall $I_{D}$ values are about one order of magnitude lower than that of the *p*-branch and show significant deviation from the linear behaviour at lower $V_{D}$ values regardless of the applied $V_{G}$. All of these observations indicate that a significantly larger barrier exists for the electron than for the hole injection from ${\mathrm{PdSe}}_{2}$ into ${\mathrm{WSe}}_{2}$. At low temperatures, within the applied $V_{D}$ range and for $V_{G}$ more than 0.5 V away from the $V_{\mathrm{th}}$, we did not observe the current saturation. However, within the same bias range at room temperature, saturation can be achieved (see Figure 2).

### 3.2. Contact Resistance of the ${\mathrm{PdSe}}_{2}$${\mathrm{/WSe}}_{2}$ Interface

The contact resistance of the interface between ${\mathrm{PdSe}}_{2}$ and ${\mathrm{WSe}}_{2}$ was evaluated independently by two approaches: parameter extraction via device modelling and direct measurements by in operando KPFM. In the first approach, we have modelled the sequence of the electrical output data by applying an equivalent electrical scheme as shown in Figure 2a (see also Section 2). The system was solved in a self-consistent manner and fitted to the set of output curves either for the hole or for the electron branch, as presented in Figure 2b,c. Parameters of the ohmic ($R_{\mathrm{ohmic}}$) and non-linear Schottky component ($I_{0}$) of the contact resistance were extracted, and width-scaled contact resistance ($WR_{C}$) was expressed considering specific points of operation (fixed $V_{D}$, $V_{G}$, and, consequently, $I_{D}$ values). We obtain $WR_{C}$ = (2.84 ± 0.53) × $10^{6}$ Ωµm for the hole branch and $WR_{C}$ = (3.72 ± 0.69) × $10^{8}$Ωµm for the electron branch. The values are reported for the operation at 300 K, with $V_{G}$ set 5 V away from the $V_{\mathrm{th}}$ in both cases of the hole and the electron branches, and under 1.5 V of source–drain bias. In particular, the need to include the non-linear Schottky element in the model is evident in a strong downward bending of the output curves at lower $V_{D}$, as pointed out by the red arrows in Figure 2c. Especially in the electron branch case, at lower $I_{D}$ the contact resistance and the entire device operation is Schottky junction-dominated, and almost all of the applied $V_{D}$ is taken by this junction as the most resistive element in the circuit. At higher $I_{D}$, the $V_{\mathrm{junction}}$ still dominates over $V_{\mathrm{ohmic}}$ by a factor of 5 to 10.

In the second approach to evaluating the contact resistance of the ${\mathrm{PdSe}}_{2}$${\mathrm{/WSe}}_{2}$ interface, we have used in operando KPFM. This technique measures the electric potential several nanometres above the channel during device operation. Therefore, it resolves the potential drops between the electrodes, and allows independent distinguishing of the potential drops that correspond to the drain (not observed in our case), the channel, and the source [16,32]. An example of the potential drop profiles is presented in Figure 2d in the hole branch on state and for varied $V_{D}$ between 0.5 V and 1.5 V. Four regions are clearly distinguishable in the potential drop profiles: flat potential values corresponding to the source and drain regions of the scan, a monotone drop of the potential along the channel, and a much steeper drop at the contact to the source electrode. Linear fits to these elements are presented by dashed black lines. The steeper drop connected to the transition between the channel-related potential drop and the source contact region is directly related to the $V_{\mathrm{junction}}+V_{\mathrm{ohmic}}$ in the device model. Knowing the $I_{D}$ values during the potential drop profile measurements and the width of the device, it is possible to express the observed junction-related potential drop as the width-scaled contact resistance.

Figure 2e presents the potential drop profiles when the connections between the source and the drain are exchanged, effectively reversing the current flow direction. We observe that the contact resistance associated potential drop is connected to the grounded source electrode, i.e., that the ${\mathrm{PdSe}}_{2}$${\mathrm{/WSe}}_{2}$ interface is rectifying. This proves the predominant Schottky nature of the contact resistance, as also suggested by the model.

Lastly, when biased under very similar operation points as in the case of the contact resistance extraction from the electrical output data sets (Figure 2f), we obtain the following device width-scaled contact resistance values obtained from in operando KPFM: $WR_{C}$ = 2.78 × $10^{6}$ Ωµm for the hole-branch and $WR_{C}$ = 3.44 × $10^{8}$Ωµm for the electron branch.

The obtained $WR_{C}$ values imply that ${\mathrm{PdSe}}_{2}$ is an effective hole injector. This is seen from the two orders of magnitude larger contact resistance of the electron branch under similar operation conditions. Furthermore, the contact resistance of the ${\mathrm{PdSe}}_{2}$${\mathrm{/WSe}}_{2}$ interface for *p*-type operation performs similar to the commonly employed evaporated metallic contacts [62,63] while preserving the intrinsic doping levels and the ambipolar nature of the ${\mathrm{WSe}}_{2}$. Reported values for ${\mathrm{WSe}}_{2}$ contact resistance range from $10^{8}$ Ω µm to $10^{5}$ Ω µm with electrostatic gating and down to $10^{4}$Ω µm for electrolyte gating that can induce very high density states in ${\mathrm{WSe}}_{2}$ [45,56,62]. Some of the lowest values reported for the contact resistance (1.1 × $10^{5}$Ω µm) are with Pt electrodes, where MIGS cannot be excluded at the electrode interface [45].

### 3.3. Optimizing Contact Interface via Laser-Driven Oxidation of ${\mathrm{WSe}}_{2}$

Recent work has shown that the application of mild oxygen plasma can be an effective way to reduce the Schottky barrier in multilayer ${\mathrm{WSe}}_{2}$ FETs [64,65,66]. The plasma treatment causes the formation of a conductive tungsten selenium oxide (${\mathrm{WSe}}_{y}$$O_{x}$). The oxide was found to form in a layer-by-layer manner [64,65], effectively generating a ${\mathrm{WSe}}_{y}$$O_{x}$${\mathrm{/WSe}}_{2}$ heterostructure that acts as a facilitator for the hole injection [66,67]. However, it is important to protect the channel active area during the plasma treatment to avoid device degradation. We wanted to investigate if a laser-based approach could open a way to achieve similar modification of ${\mathrm{WSe}}_{2}$, as with the mild plasma treatment. An advantage of the laser-driven oxidation approach is straightforward patterning by laser scanning. Using laser irradiation (532 nm, 50 mW) under ambient conditions, we have observed a similar oxidation process of ${\mathrm{WSe}}_{2}$.

**Figure 3:**
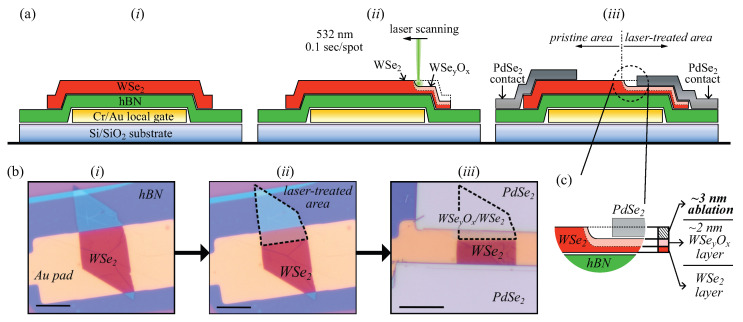
Laser treatment of ${\mathrm{WSe}}_{2}$: ((**a**) *i*–iii) Schematic cross-section of the laser-treated devices (not to scale), presenting the laser treatment process of the electrode interface step by step. ((**b**) *i*–iii) Optical micrographs (scale 5 µm) of a representative device corresponding to each fabrication step in ((**a**) *i*–iii). ((**a**,**b**) *i*) The heterostack of ${\mathrm{WSe}}_{2}$/hBN on a local gate electrode prior to the laser treatment, and ((**a**,**b**) ii) after the top part of the ${\mathrm{WSe}}_{2}$ flake was scanned by the laser (exposed part of the ${\mathrm{WSe}}_{2}$ flake is indicated by the dashed lines). ((**a**,**b**) iii) The same device after stamping of ${\mathrm{PdSe}}_{2}$ contacts. In the presented case, only one side of the channel–electrode interface was laser-treated. (**c**) A zoom in on the schematic in ((**a**) iii) highlighting the part of the ablated ${\mathrm{WSe}}_{2}$ layer, part of the oxidised ${\mathrm{WSe}}_{y}$$O_{x}$ layer, and the unmodified part of the ${\mathrm{WSe}}_{2}$ layer.

To explore the influence of the ${\mathrm{WSe}}_{y}$$O_{x}$ layer on the contact properties between ${\mathrm{WSe}}_{2}$ and ${\mathrm{PdSe}}_{2}$, we have irradiated an area of the ${\mathrm{WSe}}_{2}$ flake that is slightly larger than the contact area with ${\mathrm{PdSe}}_{2}$. After the laser treatment, ${\mathrm{PdSe}}_{2}$ flakes were transferred and used as contacts. Figure 3a(*i*–iii) show the schematic representation of the laser treatment and the device assembly process for the ${\mathrm{WSe}}_{y}$$O_{x}$-modified contacts. Figure 3b(*i*–iii) represent the corresponding optical images of the flake and the final device, where only one side of the flake was treated by the laser. Figure 3c presents a zoomed-in region of the interface to highlight the parts of the ablated layers, oxidised layers, and remaining pristine ${\mathrm{WSe}}_{2}$ layers.

**Figure 4:**
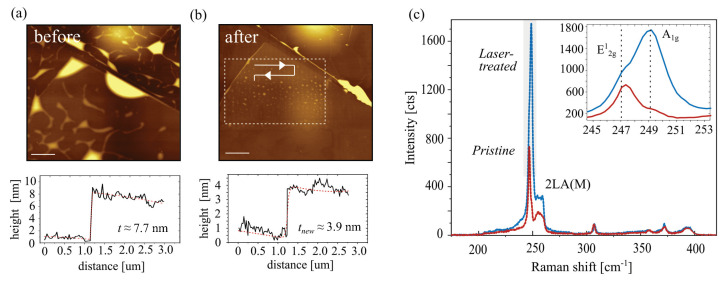
Topography changes and Raman investigation of laser-treated ${\mathrm{WSe}}_{2}$: (**a**) Atomic force microscopy (AFM) image of a ${\mathrm{WSe}}_{2}$ flake on hBN before laser exposure with the corresponding line profile (height) of the flake. The predominant morphological features are water/air bubbles trapped at the${\mathrm{WSe}}_{2}$/hBN and ${\mathrm{hBN/SiO}}_{2}$ interfaces. (**b**) The same area as in (**a**) treated with a 50 mW 532 nm laser beam. The exposed region is marked with a dashed rectangle, and the laser scanning direction is indicated with an arrow. The corresponding height profiles are presented at the bottom of the topography images. (**a**,**b**) Lateral scale bar 2 µm, z-scale 25 nm. (**c**) Raman spectrum before and after laser irradiation, recorded with 5 mW, 532 nm, and 5 × 10 s acquisition parameters. The main ${\mathrm{WSe}}_{2}$ peaks are preserved and enhanced in intensity after the laser treatment. Inset (**b**) presents a zoomed-in region of the main ${E^{1}}_{2g}$ and $A_{1g}$ modes.

AFM was performed to observe the morphological and height changes due to laser treatment. The results are presented in Figure 4a,b. AFM image before laser treatment shows large bubbles formed at the interface between ${\mathrm{WSe}}_{2}$ and hBN, as well as between hBN and ${\mathrm{SiO}}_{2}$ interface. This is expected for 2D material heterostructures assembled under ambient conditions and using PDMS stamps due to the entrapment of air and water [68,69,70]. Such interfaces result in localised charge-trap and scattering centres, and a flat interface is desired to achieve better performance [71,72]. Interestingly, laser treatment resulted in the removal and migration of these bubbles from the scan area, even at the regions not directly exposed to the laser irradiation. This is illustrated in Figure 4b. Such behaviour can be attributed to the self-cleaning property of 2D materials under a systematic sweep of the laser spot which allows local heating and migration of the trapped water/air bubbles at the interfaces [71,72]. The arrows in Figure 4b represent the direction of laser sweeping, and the dashed rectangle indicates the laser-exposed area. Figure 4a,b (bottom) show the change in height of the flake before and after the treatment. The resultant height corresponds to a thickness of 3.9 nm. This indicates the ablation of about five mono-layers of ${\mathrm{WSe}}_{2}$, and the remaining flake effectively forms a ${\mathrm{WSe}}_{y}$$O_{x}$${\mathrm{/WSe}}_{2}$ heterostructure. Combined with Raman spectroscopy data (Figure 4c) we estimate that after the laser treatment, about three layers of ${\mathrm{WSe}}_{2}$ remain, with about 2 nm of ${\mathrm{WSe}}_{y}$$O_{x}$ formed on top [64,65,66]. Raman spectroscopy was also performed to verify the crystal quality of the laser-modified flakes. Figure 4c presents the Raman spectra before and after the laser treatment of a ${\mathrm{WSe}}_{2}$ flake. The increase in the Raman intensity of the $A_{1}g$ mode (shown in the inset) after the treatment validates the thinning of ${\mathrm{WSe}}_{2}$ with the oxidation of top layers. Such an increase in the intensity of the peaks is related to the thinning of ${\mathrm{WSe}}_{2}$ and an increase in the phonon lifetime [45,73]. An increase in phonon lifetime should be also observed in the according change in the device-apparent field-effect mobility. However, for both treated and the untreated devices, the apparent hole mobilities were within the sample-to-sample variation.

### 3.4. Electrical Characteristics of ${\mathrm{WSe}}_{2}$ FETs with ${\mathrm{WSe}}_{2}$${\mathrm{/WSe}}_{y}$$O_{x}$${\mathrm{/PdSe}}_{2}$ Electrode Interface

**Figure 5:**
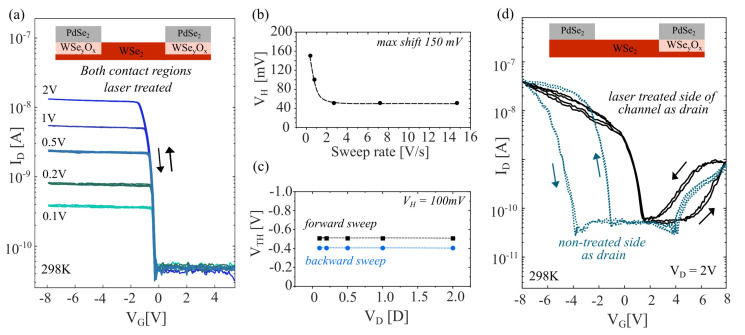
Electrical response of ${\mathrm{WSe}}_{2}$${\mathrm{/WSe}}_{y}$$O_{x}$${\mathrm{/PdSe}}_{2}$ electrode interface: (**a**) Semi-log scale electrical transfer curves of a ${\mathrm{WSe}}_{2}$ device with both source and drain electrode interfaces treated by a laser prior to stamping ${\mathrm{PdSe}}_{2}$ contacts measured at 298 K, 2 × ${\mathrm{10}}^{-2}$ mbar. (**b**) $V_{H}$ plot as a function of a scan speed (measured at 298 K, 2 × $10^{-2}$ mbar). (**c**) Position of the $V_{\mathrm{th}}$ for both forward and backward $V_{G}$ sweeping with varied $V_{D}$. The difference indicates the hysteresis ($V_{H}$) is independent of $V_{D}$. (**d**) Semi-log scale electrical transfer curves for an asymmetric ${\mathrm{WSe}}_{2}$ FET with only one contact pad treated by the laser. The dotted lines represent the drain electrode connected to the non-treated ${\mathrm{PdSe}}_{2}$ contact side, while source and drain were swapped for the solid black line. The arrows indicate the $V_{G}$ sweeping direction.

Figure 5a represents the electrical transfer curves for the ${\mathrm{WSe}}_{2}$ device with both source and drain electrode interfaces modified by the laser treatment. The device showed a notable decrease in $I_{\mathrm{ON}}$. However, highly stable *p*-type devices were realised with respect to $V_{\mathrm{th}}$ variations between subsequent sweeps at room temperature operation and also under varied $V_{G}$ sweeping rates. $V_{\mathrm{th}}$ for the hole branch of the ${\mathrm{WSe}}_{y}$$O_{x}$-modified contacts was found to be at (−0.42 ± 0.06) V, which is about four times lower than the ${\mathrm{PdSe}}_{2}$${\mathrm{/WSe}}_{2}$ interface. More importantly, the sample-to-sample-, forward/backward sweep-, and multiple sweep-related variations in the $V_{\mathrm{th}}$ value are almost completely reduced. The dominant *p*-type behaviour with the quenching of the electron branch can be associated with ${\mathrm{WSe}}_{y}$$O_{x}$, which acts as an efficient hole injection layer [66,67]. The ${\mathrm{WSe}}_{y}$$O_{x}$${\mathrm{/WSe}}_{2}$ layer also extends beyond the contact regions into the channel (for about 1 µm) to ensure that the contact is not made directly with the unmodified ${\mathrm{WSe}}_{2}$. Consequently,${\mathrm{WSe}}_{y}$$O_{x}$ could also introduce interface traps in the channel active area. To test this, we have probed the stability of the devices by examining the hysteresis voltage ($V_{H}$), as a difference in the $V_{\mathrm{th}}$ between the forward and backward sweeping electrical transfer curves. $V_{H}$ values for the varied $V_{G}$ sweep rates are shown in Figure 5b. The device remained stable at high sweeping rates (up to 15 V/s) with a negligible hysteresis of 55 mV. An increase in the hysteresis of up to 150 mV was noted at low sweeping rates. The hysteresis values correspond well to the pristine ${\mathrm{PdSe}}_{2}$${\mathrm{/WSe}}_{2}$${\mathrm{/PdSe}}_{2}$ devices, indicating that the ${\mathrm{WSe}}_{y}$$O_{x}$ layers did not affect device stability. The observed hysteresis is likely related to the interface between hBN and ${\mathrm{WSe}}_{2}$ or is inherent to the ${\mathrm{WSe}}_{2}$ layers.

Two-dimensional semiconductors commonly show large variations in the $V_{\mathrm{th}}$ at varying drain voltages which also impacts the device stability [6,74,75]. To test this, we subjected our device to a $V_{D}$ ranging from 0.5 V to 2.0 V. The device maintained the same $V_{\mathrm{th}}$ for forward and backward sweeps with a $V_{D}$-independent hysteresis of 98 mV. The subthreshold swing (SS) values were also significantly improved from 200 mV/dec for the non-treated to 100 mV/dec for the treated devices. It is worth mentioning that these devices operated without a top encapsulation and therefore, a high-k dielectric encapsulation, optimization of the hBN thickness, and integration into dual-gate geometries can further improve ${\mathrm{WSe}}_{2}$ FET performance. The obtained SS values, especially for the laser-treated devices, perform better than commonly reported back-gate implemented FETs, where the SS values range from 2500 mV/dec to 400 mV/dec [11,18,20]. Some of the lowest reported SS values for ${\mathrm{WSe}}_{2}$ devices (95 mV/dec) were achieved by utilizing 20 nm of ${\mathrm{HfO}}_{2}$ as the gate insulator and *p*-branch matching Pt contacts [45].

We have also fabricated asymmetric devices where only one side of the channel was treated with the laser (as also shown in Figure 3). An example of the electrical transfer curves observed for such devices is presented in Figure 5d. A large hysteresis window was observed, which directly depends on the choice of the drain electrode, i.e., the device exhibits rectifying behaviour with respect to the induced hysteresis. For the case where the laser-treated region was used as a drain, a stable *p*-branch was realised with negligible hysteresis, represented by a solid black curve in Figure 5d. A pronounced hysteresis of 2 V was only present for the *n*-branch. This was observed to be in stark contrast to the behaviour of the same device when the non-treated region was used as the drain. In that case, a *p*-branch hysteresis of 4 V was observed. In both cases, the hysteresis was stable for multiple forward–backward sweeps as indicated by the arrows. Such behaviour can be associated with large differences between the number of carriers available underneath the contact regions. As one end of the channel is intentionally favouring hole injection and prohibiting electron injection, the other stays in its original form. Such large and stable rectifying hysteretic behaviour could be employed in novel concepts as computing in memory and self-reconfiguring electronics [76,77].

## 4. Conclusions

In summary, we have introduced ${\mathrm{PdSe}}_{2}$ contacts to ${\mathrm{WSe}}_{2}$ FETs that enable effective hole injection, enhanced *p*-type performance, and preserve the intrinsic ambipolar response of ${\mathrm{WSe}}_{2}$. ${\mathrm{PdSe}}_{2}$ contacts allow essentially hysteresis-free electrical response while maintaining high on-state currents and $I_{\mathrm{ON}}/I_{\mathrm{OFF}}$ ratio enhancement by one order of magnitude in comparison to graphite-contacted devices. Considering the low temperatures required for the ${\mathrm{PdSe}}_{2}$ growth, it is a promising electrode candidate especially when considering the potential that ${\mathrm{PdSe}}_{2}$ brings for the upscaling of 2D-material-based electronics and the incorporation of ambipolar ${\mathrm{WSe}}_{2}$ to post-CMOS architectures.

We extended the study to also contact laser-treated ${\mathrm{WSe}}_{2}$, where the laser irradiation induces the formation of a conductive ${\mathrm{WSe}}_{y}$$O_{x}$ layer at the electrode interface. In this case, we observed highly stable *p*-type behaviour of the devices with a two-fold improvement in the subthreshold swing, stabilization of the $V_{\mathrm{th}}$ for the hole branch. Interestingly, if only one electrode interface is modified by the laser treatment, asymmetric ${\mathrm{WSe}}_{2}$ FETs were achieved, which exhibited pronounced and stable hysteretic behaviour of only one (electron or hole) branch. The hysteresis was dependent on the direction of applied drain voltage. Such device response can be used to design in-memory computing and reconfigurable electronic concepts based purely on 2D interfaces.

## Data Availability

The data generated within this study and the samples related to this study are available from the corresponding author upon reasonable request.
